# 4528 Sirtuin 3 activation as a potential renoprotective therapy in a mouse model of Alport syndrome

**DOI:** 10.1017/cts.2020.91

**Published:** 2020-07-29

**Authors:** Bryce Jones, Komuraiah Myakala, Xiaoxin Wang, Andrew Libby, Shogo Takahashi, Kanchan Bhasin, Suman Ranjit, Avi Rosenberg, Moshe Levi

**Affiliations:** 1 Georgetown - Howard Universities; 2 Georgetown University; 3 Johns Hopkins University

## Abstract

OBJECTIVES/GOALS: Sirtuin 3 (Sirt3), a mitochondrial NAD^+^-dependent deacetylase, is decreased in diverse models of kidney disease, and Sirt3 activation prevents disease progression in many of those models. We are investigating if pharmacological activation of Sirt3 ameliorates kidney disease in a mouse model of Alport syndrome. METHODS/STUDY POPULATION: Alport syndrome is a hereditary orphan disease arising from a defect in the collagen IV α3α4α5 heterotrimer, a component of the glomerular basement membrane. Male and female Col4a3^tm1Dec^ knockout mice and wild type controls on the 129X1/SvJ background were harvested at 9–10 weeks of age. Serum and urine were collected prior to euthanasia; renal pathology was assessed by histology; and renal cortical mRNA and protein levels were assessed by qRT-PCR and western blot, respectively. Studies are ongoing using dietary administration of a Sirt3 activator, nicotinamide riboside (500 mg/kg/day), in Col4a3 transgenic mice on both the 129X1/SvJ and C57BL/6J backgrounds. RESULTS/ANTICIPATED RESULTS: Col4a3^−/−^ mice have elevated BUN (P < 0.0001, both sexes), serum creatinine (P < 0.001, male; P < 0.0001, female), and urinary albumin-to-creatinine ratio (P < 0.0001, both sexes) compared to Col4a3^+/+^ controls. On histology, Col4a3^−/−^ mice have extensive renal fibrosis compared to Col4a3^+/+^ controls. Sirt3 expression is decreased in the renal cortices of Col4a3^−/−^ mice at the mRNA (P < 0.0001, male; trend, P = 0.07, female) and protein levels (P < 0.05, male; P < 0.001, female) compared to Col4a3^+/+^ controls. All experiments had 5–9 mice per group. Results of the prevention study with nicotinamide riboside, a Sirt3 activator, are unknown at the time of abstract submission. DISCUSSION/SIGNIFICANCE OF IMPACT: Col4a3^−/−^ mice have severe renal impairment and decreased renal cortical expression of Sirt3 at the mRNA and protein levels compared to Col4a3^+/+^ controls. However, it is unknown at this time if pharmacologically activating Sirt3 prevents this renal decline.

